# The renal artery-aorta angle associated with renal artery plaque: a retrospective analysis based on CT

**DOI:** 10.1186/s12880-023-00997-5

**Published:** 2023-03-25

**Authors:** Hongzhi Yang, Ruwu Yang

**Affiliations:** Department of Radiology, XD Group Hospital, Xi’an, 710077 Shaanxi China

**Keywords:** Renal artery plaque, Renal artery and vein, 320-row CT

## Abstract

**Purpose:**

To investigate the relationship between renal artery anatomical configuration and renal artery plaque (RAP) based on 320-row CT.

**Methods:**

The abdominal contrast-enhanced CT data from 210 patients was retrospectively analyzed. Among 210 patients, there were 118 patients with RAP and 92 patients with no RAP. The anatomical parameters between lesion group and control group were compared and analyzed by using t-test, χ2-test and logistic regression analysis.

**Results:**

(1) There were statistical differences on age, hypertension, diabetes, hypertriglyceridemia and hypercholesterolemia between lesion group and control group. (2) The differences on the distribution and type and of RAP between lesion group and control group were statistically significant. The most common position was the proximal, and the most common type was calcified plaque. (3)There were significant statistical differences on the proximal diameter of renal artery and renal artery-aorta angle A between lesion group and control group. The differences on the other anatomical factors between two groups were not statistically significant. (4) The result of logistic regression analysis showed that right RAP was related to age, hypertension and right renal artery angle A (the AUC of ROC = 0.82), and left RAP was related to high serum cholesterol, age and left renal artery angle A(the AUC of ROC = 0.83). (5) The RAP was associated with renal artery-aorta angle A, but the differences on distribution, type stability of RAP between R1 (L1) group and R2 (L2) group were not statistically significant.

**Conclusions:**

The RAP was associated with age, hypertension, hypercholesterolemia and renal artery-aorta angle A. Adults which had the greater renal artery-aorta angle A and the other above risk factors may be at increased risk for RAP.

## Introduction

Renal artery plaque (RAP) is associated with renal artery stenosis(RAS) and is known as the predominant cause of RAS, which can result in renal resistant hypertension, ischemic nephropathy, renal artery angina, chronic kidney disease, renal dysfunction, recurrent pulmonary edema, angina colic, acute left heart failure and acute coronary syndrome [1, 2]. As a indicator of atherosclerosis, RAP not only reflects the stage of progression and severity of systemic atherosclerosis, but also plays important role on prevention of coronary heart disease and acute stroke [2, 3]. RAP rupture can lead severe stenosis or occlusion of renal artery that could cause renal infarction or kidney dysfunction [4]. One study have demonstrated that patients with RAP are more likely to appear acute coronary syndrome and acute stoke than people with no RAP [5]. Takumi et al. [6] found that RAP may influence renal function after renal artery intervention. Furthermore, atherosclerotic state from donor and recipient, other anatomical and technical factors can result in transplant renal artery stenosis (TRAS), which can induce graft failure and ischemic nephropathy [7–9]. In sum, RAP makes a great unfavorable impact on human health. Therefore, it is necessary to study the risk factors influencing the formation of plaque.

Up to date, the researches related with RAP mainly focus on RAP associations with subclinical disease, RAP relevance with risk factors, RAP relationship with the progression of systemic atherosclerosis, few research on RAP associations with anatomical factors [10, 11]. There are many factors that can influence the formation of RAP**,** including traditional atherosclerosis risk factors: age, male gender, body mass index (BMI), smoking, hypertension, diabetes, carotid artery intima-media thickness, which were considered as a useful noninvasive marker of subclinical atherosclerosis [3, 5]**.** In a study, Tolkien et al. [12] found a significant association between RAP and age, gender, hypercholesterolemia and hypertension, while Siegel et al. [13] concluded that calcifications of the RAP were not related to hypertension through retrospectively analysis on abdominal CT image data. In addition, children with known risk factors, including genetic diabetes, hyperlipidemias, and renal diseases, are at higher risk for atherosclerotic plaques in adolescence [14]. However, it remains unknown as to whether RAP is associated with experimental indexes related to renal function or not. Zhun et al. [15] indicated the atherosclerotic RAS was related with regional vascular geometry, but the quantitative analysis of correlation between RAP and the specific anatomical indexes of renal vessel was not studied. Vlach Giannis Nikolaos et al. [16] showed that RNA-mediated inflammation affected the process of atherosclerotic cardiovascular disease and stability of artery plaque. Wen et al. [17] indicated that serum cystatin C may contribute stability of plaques in normal renal function and serum cystatin C was a risk predictor of plaques in mildly impaired renal function. Although the conclusion is quite controversial, there have been many studies arguing the relationship between RAP and demography or metabolic abnormalities. Only few research is available regarding the correlation between RAP and anatomic configuration of renal arteries or veins.

In addition to, it was found that some older patients with multiple underlying metabolic diseases had no RAP, but some younger patients with no underlying metabolic diseases had RAP in the daily-routine practice, which may be speculated that RAP was connected with renal vascular anatomical configuration. In view of the above facts, this study mainly investigates whether RAP is associated with anatomical factors of renal artery and vein or not, through retrospectively analyzing abdominal contrast-enhanced CT image characteristics. The common examinations related with RAP research contain multi-detector row computed tomography (MDCT), intravascular ultrasound (IVUS), and MRI [18–21]. MDCT is the most widely used owing to clearly display lumen and have a higher sensitivity of RAP. Consequently, this study does relevant research on RAP by analysis of CT image data.

## Materials and methods

### Study design and participants

**Fig. 1:**
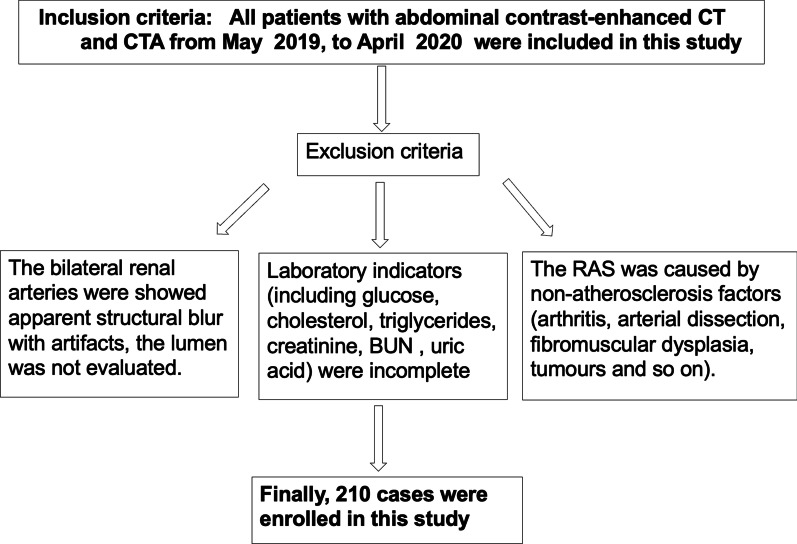
A flow diagram of the patient selection process

We retrospectively assessed consecutive patients who underwent abdominal contrast-enhanced CT for screening and evaluating- at a single tertiary center between May 2019 and April 2020. Inclusion criteria: (a) patients with abdominal CT enhancement or CTA from May, 2019, to April, 2020; (b) patients with abdominal CTA from May, 2019, to April, 2020. Exclusion criteria: (a)The bilateral renal arteries were showed apparent structural blur with artifacts, so that the lumen was not evaluated; (b). Laboratory indicators (including glucose, cholesterol, triglycerides, creatinine, BUN, uric acid) were incomplete; (c). The RAS was caused by non-atherosclerosis factors (arthritis, arterial dissection, fibromuscular dysplasia, tumors and so on). A total of 210 patients were finally enrolled in this study, and demographic and clinical data were retrospectively collected from the electronic medical records (Fig. 1). There were 108 male (43%) and 102 female (57%)with an average age of 64.1 ± 11 years old. The lesion group was defined as patients with left or right artery plaque, and the control group was defined as patients without left and right artery plaque. The lesion group was divided into two subgroups (left/right) according to the left or right renal artery of RAP. Right lesion group was divided into R1 and R2 subgroups according to the right renal artery angle A (R1 group if angle A < 61.1° and R2 group if angle A ≥ 61.1°). Left lesion group was divided into L1 and L2 subgroups according to the left renal artery angle A (L1 group if angle A < 57.9° and R2 group if angle A ≥ 57.9°). Hypertension was defined as systolic blood pressure≧140 mmHg or diastolic blood pressure above≧90 mmHg. Diabetes can be diagnosed (Fasting blood glucose is ≧ 7.0 mmol/L, or, random blood glucose and blood glucose of two hours of oral glucose tolerance test are **≧** 11.1 mmol/L). Hypercholesterolemia, hypertriglyceridemia, high serum creatinine, high-BUN and high-uric acid were defined as higher than the upper limit of the laboratory's normal reference range (cholesterol 2.48–5.17 mmol/L, triglycerides 0.57–1.70 mmol/L, creatinine 62–106 μmoI/L, BUN 2.1–7.1 mmol/L, uric acid 2.48–5.17 mmol/L). People who have smoked continuously or cumulatively for 6 months or more were smokers. Abdominal contrast-enhanced CT images demonstrated whether the RAP exits or not and showed the position and property of RAP (2.3). This study was approved and consented by the Ethics Committee of Xidian Group Hospital, and written informed consent was obtained from all participants.

### CT scanning protocol

All abdominal contrast-enhanced CT examinations were performed with a 320-slice scanner (Cannon, Japan). These examinations were performed with prior placement of a 20-gauge venous access in a median cubital vein of the right arm and after administration of 80–90 mL of nonionic iodinated contrast medium (Iodine 370 mg I/mL), followed by injection of 30 mL of saline solution at a rate of 3.5 mL/s with the use of a dual-head injector (Irich, Medical). Optimal delay was performed with the automatic intelligent bolus-tracking technique (The tracer was placed in the proximal segment of abdominal aorta with threshold 320HU). Images were acquired with the following protocol: 100–120 kV, automated mA, pitch of 1.5, slice thickness of 0.5 mm, reconstruction interval of 0.5 mm, matrix of 512 × 512, and the total scan time of 182–185 s, the scan range was from the top of the diaphragm to the lower level of the kidney and beyond in a cranio-caudal direction. The dose of radiation was 3 ~ 5 mSv.

### CT image analysis

**Fig. 2:**
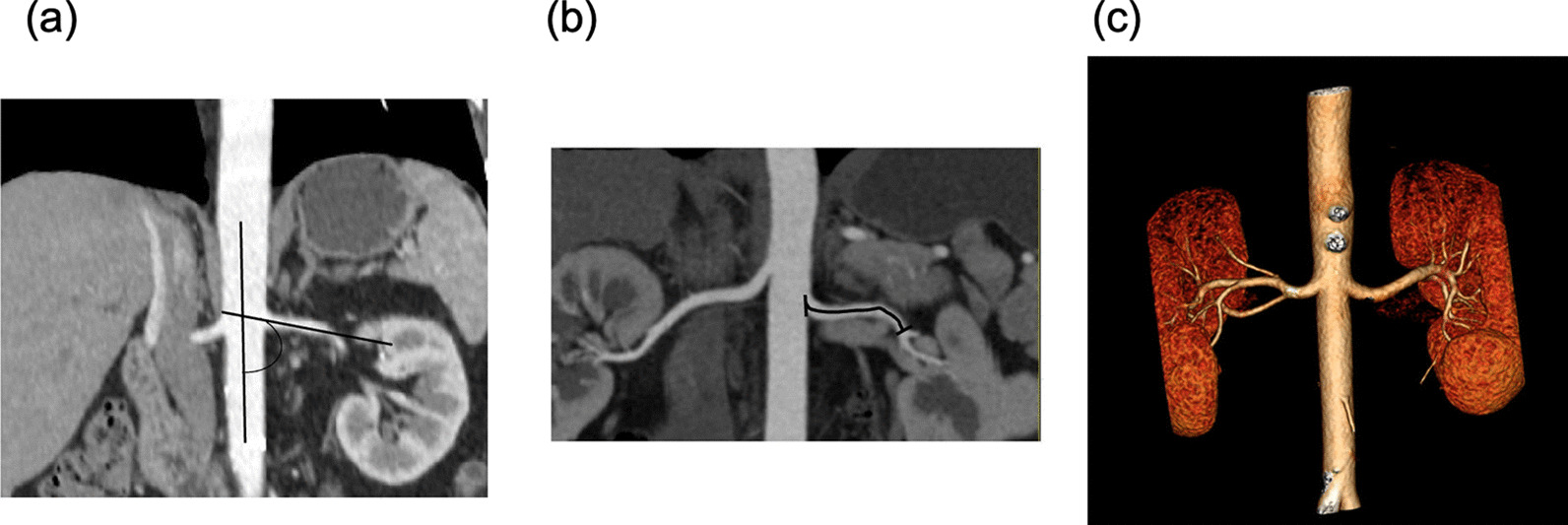
Renal artery reconstruction image. (**a**) cor-MPR, left renal artery angle A (between left renal artery and the abdominal aorta); (**b**) The length of left renal artery trunk (from the origin to the bifurcation); (**c**) Renal artery VR, which shows the origin position, route, lumen and anatomical variation of renal artery

**Fig. 3:**
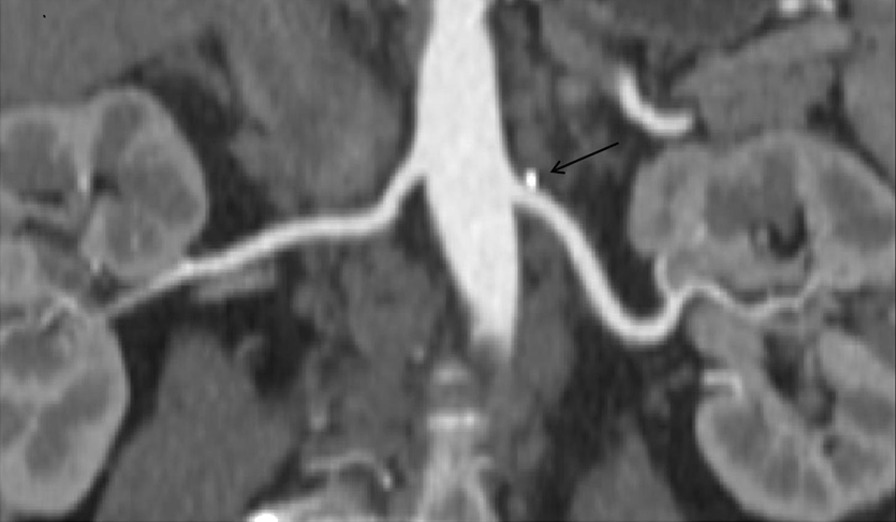
A participant (age range between 55 and 65 years old) with hypercholesterolaemia and normal blood pressure, renal angle A (right 41.64°, left 46.56°), cor-CPR shows that there is the calcified plaque in the proximal left renal artery and there is no RAP in the right renal artery

**Fig. 4:**
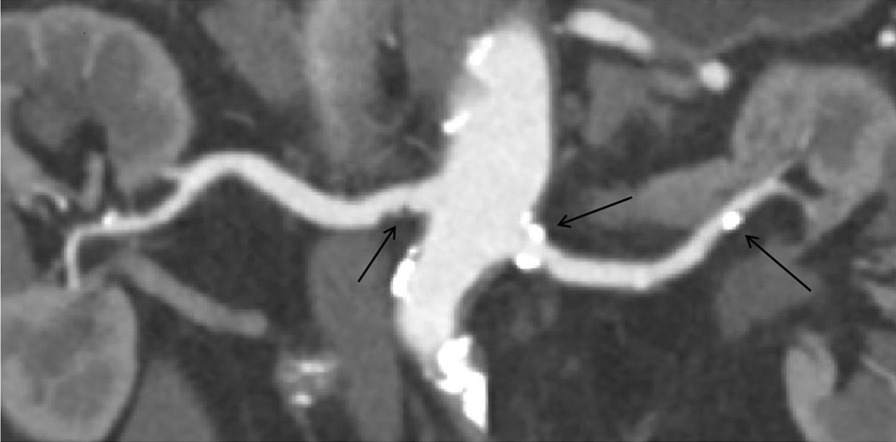
A participant (age range between 80 and 85 years old) with hypertension and hypercholesterolaemia, renal angle A (right 65.86°, left 62.16°), cor-CPR shows there is the calcified plaque in the proximal and the distal left renal artery and there is the non-calcified plaque in the proximal right renal artery

All the abdominal contrast-enhanced CT original data were transmitted to an independent imaging workstation (Cannon Vistra6.5, Japan) for post-processing. The primary methods of imaging processing were maximum intensity projection, multi-planar reconstruction, curved-planar reconstruction, and volume rendering [22]. Coronal multi-planar reconstruction (MPR, Fig. 2a) with a 3-mm thickness, Coronal curved planar reconstruction (CPR, Fig. 2b) and volume rendering (VR, Fig. 2c) were used for the evaluation of renal artery. The CT original imaging data were evaluated and analyzed by radiologists with ten years of experience. The position of plaques was documented as the following criteria: (a) the proximal section was defined as proximal 1/3 segment of renal artery trunk; (b) the middle section was defined as middle 1/3 segment of renal artery trunk; (c) the distal section was defined as distal 1/3 segment of main renal artery; (d) the diffuse was defined as two or more segments. The type of the plaque was assessed as the following criteria: calcified plaques–CT value ≥ 130HU, non-calcified plaques–CT value < 130HU, mixed plaques–both non-calcified and calcified plaques [23, 24]. Stable plaque includes calcified plaque and mixed plaque. Unstable plaque includes non-calcified plaque. The diameter of renal artery (proximal, mid, distal) was measured in the axial image. In addition, the angle between the renal artery and the abdominal aorta (angle A) was measured in the coronal MPR image, the angle between the proximal 1/3 segment and the distal 1/3 segment or the rise of the most curved angle (angle B) was measured in the transverse or coronal MPR image, the distance of the bilateral renal artery origin position was measured in the coronal MPR image, the length of the main trunk was measured in the CPR image (Fig. 2b), the vascular variation include accessory renal artery or premature branch (length of the main trunk < 15 mm in CPR), the above parameters were documented by the two radiologists with ten years of experiences. They resolved inconsistent measurement results through repeated measurement (Figs. 3 and 4).

### Statistical analyses

Quantitative data are expressed as mean value ± standard deviation, and qualitative data are expressed as percentage. Statistical methods included the chi-square test, two-sample t-test and logistic regression analysis. The chi-square was used to evaluate the distribution trend of RAP, the proportion of various plaques, the correlation between RAP and renal artery variation, the association between RAP and clinical variables, the difference on the incidence of RAP between the different the renal artery angle A. The two-sample t-test was applied to examine statistical differences on anatomical parameters between groups. Logistic regression analysis was carried out to investigate whether the above statistically different factors are related to the RAP or not. All statistical analyses were performed by using SPSS software (version 25.0). The p-value less than 0.05 were considered statistically significant in this study (FDR corrected).

## Results

### Comparison of clinical information

A total of 210 subjects were included in this study (48.6% female).The average age was 64.1 ± 13.8 years old. Among all patients, 118 patients had RAP, and 92 patients had no RAP. There were significant statistical differences on age, hypertension, hypercholesterolemia, hypertriglyceridemia, and diabetes between the lesion group and control group. The other clinical factors of renal artery between two groups were not significant statistical (*p* < 0.05 Table 1). Detailed clinical information about the study population was summarized in Table 1.

**Table 1:** Cohort characteristics stratified by the presence of renal artery plaque

	Total(n = 210)	Lesion group (n = 118)	Control group(n = 92)	*p*-value
Basic information				
Gender, man (%)	108(51.4)	59(50.0)	49(55.7)	0.74
Age, yeas old	64.05 ± 13.8	71.6 ± 9.5	54.4 ± 12.4	< 0.01^**ab**^
Clinical symptoms				
Kidney disease, n (%)	80(38.1)	45(38.1)	35(38.0)	1
High risk factors				
Hypertension, n (%)	81(38.6)	59(50.0)	22(23.9)	< 0.01^**a**^
Hypercholesterolemia, n (%)	67(31.9)	47(39.8)	20(21.7)	< 0.01^**b**^
Hypertriglyceridemia, n (%)	51(24.3)	44(37.3)	7(7.6)	< 0.01
Diabetes, n (%)	30(14.3)	26(22.0)	4(4.3)	< 0.01
Smoke, n (%)	108(51.4)	10(8.5)	4(4.3)	0.31
Kidney function index				
High-serum creatinine, n (%)	85(40.5)	49(41.5)	36(39.1)	0.83
High-urea, n (%)	55(26.2)	33(28.0)	22(23.9)	0.61
High-uric acid, n (%)	47(22.4)	24(20.3)	23(25.0)	0.52

Logistic regression analysis of the above statistical different indicators shows that right renal artery plaque is related to age, hypertension (^a^). Left renal artery plaque is related to age, hypercholesterolaemia (^b^)

### Comparison of anatomical factors

#### Comparison of distribution and type of RAP

1. There was no statistical difference on the incidence of RAP between unilateral side and bilateral side(*p* > 0.05). The incidence of RAP between left renal artery and right renal artery were approximate with no statistical difference(*p* > 0.05).
2. There was no statistical difference on the distribution and type of RAP between left renal artery and right renal artery (*p* > 0.05).The differences on the distribution and type of RAP from left/right renal artery were significant statistical different(*p* < 0.05).The RAP was found more frequently at the proximal section of renal artery. The calcified plaque was the most common. Detailed information about the RAP was summarized in Table 2.

**Table 2:** Comparison of characteristic of left and right renal artery plaque

	Right renal artery	Left renal artery	*P*-value
			(*P*1, *P*2, *P*1-2)
Renal artery plaque	88(41.9%)	94(44.8%)	0.55(*P*1-2)
Distribution			
Proximal	63(71.6%)	67(71.3%)	< 0.01, < 0.01, 0.18
Middle	21(23.9%)	18(19.1%)	
Diatal	3(3.4%)	2(2.1%)	
Diffuse	1(1.1%)	7(7.4%)	
Type			
Calcified plaque	49(55.7%)	47(50.0%)	< 0.01, < 0.01, 0.31
Non-calcified plaque	12(13.6%)	21(22.3%)	
Mixed plaque	27(30.1%)	26(27.7%)	
Accessory renal artery			
Lesion group	15(17.0%)	9(10.2%)	0.67(*P*1), 0.14(*P*2)
Control group	17(13.9%)	18(14.8%)	
Premature branch			
Lesion group	34(38.6%)	40(45.5%)	0.13(*P*1), 0.51(*P*2)
Control group	51(41.8%)	43(35.2%)	

*P*1–-comparison within the right renal artery group

*P*2–-comparison within the left renal artery group

*P*1-2–-comparison between left and right renal artery groups

#### Comparison of anatomical factors of renal artery

**Fig. 5:**
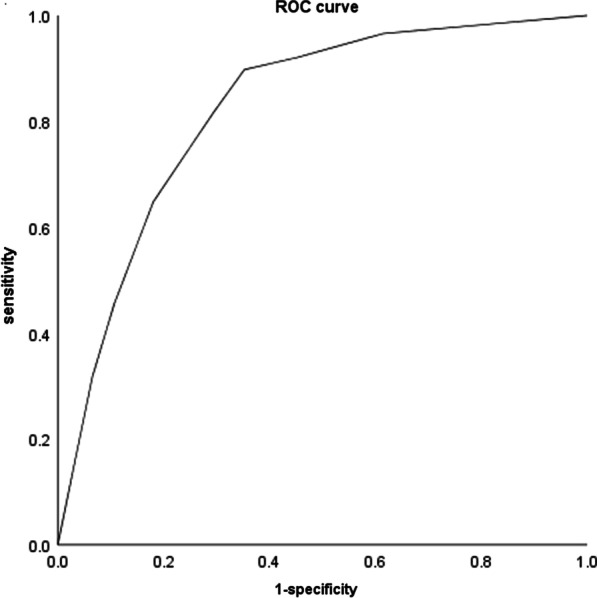
Right renal artery plaque associations with age, hypertension and right renal artery angle A (ROC curve, AUC = 0.82)

**Fig. 6:**
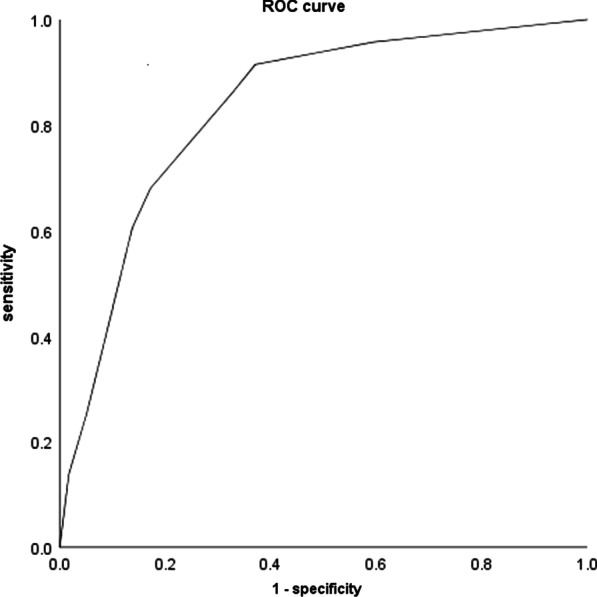
Left renal artery plaque associations with age, hypercholesterolaemia and left renal artery angle A (ROC curve, AUC = 0.83)

1. The difference on the proximal diameter of the renal artery and renal artery angle A between lesion group and control group was significant statistical (*p* < 0.05 Tables 3 and 4). The other anatomical factors of renal artery between two groups were not significant statistical(*p* < 0.05 Tables 2, 3 and 4).
2. Logistic regression analysis found that right RAP was significantly related to age, hypertension and right renal artery angle A, and left RAP was related to age, hypercholesterolemia and left renal artery angle A (Tables 1, 2, 3 and 4). The AUC of ROC (right RAP) is 0.82 (Fig. 5), and the AUC of ROC (left RAP) is 0.83 (Fig. 6).
3. The difference on the distribution, type and stability of RAP between R1 and R2 group (between L1 and L2 group) were not statistically significant (*p* > 0.05 Tables 5 and 6). The renal artery angle A had no significant effect on the distribution, type and stability of RAP.

**Table 3:** Comparison of anatomical factors between lesion group and control group (right renal artery)

	Control group (n = 122)	Lesion group (n = 88)	t-value	p-value
Renal artery diameter (mm)				
Proximal	5.30 ± 1.14	4.91 ± 1.01	2.57	0.01*
Middle	3.85 ± 0.85	3.76 ± 0.89	0.68	0.5
Distal	3.81 ± 0.92	3.71 ± 1.01	0.71	0.48
Average	4.29 ± 0.87	4.16 ± 0.89	1.04	0.3
Renal artery angle(°)				
Angle A	54.53 ± 17.07	60.14 ± 14.70	2.55	0.01*^a^
Angle B	130.26 ± 31.41	131.87 ± 27.62	0.39	0.7
Renal artery opening distance(mm)	7.68 ± 1.14	6.66 ± 1.14	1.44	0.15
The main trunk length(mm)	41.51 ± 13.73	41.73 ± 13.21	0.12	0.9
Renal vein diameter(mm)				
Proximal	6.30 ± 2.05	5.99 ± 2.57	0.94	0.35
Middle	6.84 ± 1.99	6.32 ± 2.14	1.79	0.08
Distal	6.32 ± 2.28	5.92 ± 2.22	1.29	0.2
Average	6.49 ± 1.74	6.17 ± 2.02	1.54	0.13

^*^p < 0.05

Logistic regression analysis of the above statistical different indicators show that right renal artery plaque is related to renal artery angle A(*a), and is not related to the proximal diameter(*)

**Table 4:** Comparison of anatomical factors between lesion group and control group (left renal artery)

	Control group (n = 116)	Lesion group (n = 94)	t-value	*p*-value
Renal artery diameter (mm)				
Proximal	5.44 ± 1.13	5.04 ± 1.03	2.71	0.01*
Middle	4.37 ± 0.90	4.33 ± 0.84	0.34	0.74
Distal	4.16 ± 0.90	4.19 ± 0.84	0.23	0.82
Average	4.66 ± 0.90	4.52 ± 0.77	1.04	0.23
Renal artery angle (°)				
Angle A	53.98 ± 15.59	62.79 ± 15.19	4.13	< 0.01*^b^
Angle B	126.53 ± 37.28	123.51 ± 34.86	0.6	0.55
Renal artery opening distance (mm)	7.28 ± 13.23	7.20 ± 13.23	0.13	0.9
The main trunk length (mm)	34.20 ± 13.23	36.09 ± 13.02	1.04	0.3
Renal vein diameter (mm)				
Proximal	7.81 ± 2.16	7.35 ± 1.79	1.7	0.09
Middle	6.77 ± 2.00	6.40 ± 1.94	1.34	0.18
Distal	4.64 ± 2.16	4.38 ± 2.22	1.29	0.39
Average	6.40 ± 1.69	6.04 ± 1.60	1.6	0.11

^*^p < 0.05

Logistic regression analysis of the above statistical different indicators show that left renal artery plaque is related to renal artery angle A(*b), and is not related to the proximal diameter(*)

**Table 5:** Comparison of distribution/type/stability of RAP within right renal artery lesion group

	R1 group (n = 55)	R2 group (n = 33)	t-value	*p-value*
Distribution				
Proximal	41(74.6%)	22(66.7%)	5.3	0.15
Middle	11(20.0%)	10(30.3%)		
Diatal	2(3.6%)	1(3.0%)		
Diffuse	1(1.8%)	0		
Type				
Calcified plaque	8(14.5%)	4(12.1%)	1.95	0.38
Non-calcified plaque	30(54.5%)	19(57.6%)		
Mixed plaque	17(31.0%)	10(30.3%)		
Stability				
Stable plaque	25(45.5%)	14(42.4%)	0.08	0.78
Unstable plaque	30(54.5%)	19(57.6%)

**Table 6:** Comparison of distribution/type/stability of RAP within left renal artery lesion group

	L1 group (n = 54)	L2 group (n = 40)	t-value	*p*-value
Distribution				
Proximal	38 (70.4%)	30(75.0%)	4.12	0.25
Middle	9 (16.7%)	8(20.0%)		
Distal	2 (3.7%)	0		
Diffuse	5 (9.2%)	2(5.0%)		
Type				
Calcified plaque	10 (18.5%)	11(27.5%)	1.47	0.48
Non-calcified plaque	28 (51.9%)	19(47.5%)		
Mixed plaque	16 (29.6%)	10(25.0%)		
Stability				
Stable plaque	26 (48.1%)	21(52.5%)	0.17	0.68
Unstable plaque	28 (51.9%)	19(47.5%)		

## Discussion

RAP was the most common cause of RAS, which can bring about unfavorable impact on human health, including renal insufficiency, renal hypertension, ischemic nephropathy, acute cardiovascular events and so on [25–29]. This study showed that people with high-risk anatomical factor (the greater renal artery angle A) and high-risk traditional factors (older age, hypertension, hypercholesterolemia) had the higher risks of RAP. It is essential to attach importance to high risk factors related to RAP. Intervening risk factors may improve the long-term prognosis of the patients to the greatest extent [30].

### Clinical information

RAP is the local indicator of systemic atherosclerosis process [2, 12]. This research has confirmed that right RAP is significantly related to age, hypertension and right renal artery angle A, and left RAP is related to age, hypercholesterolemia and left renal artery angle A (Figs. 3 and 4). The possible reasons for this difference are as follows: First. There may exist anatomical and histological slight differences on the structures of wall between left and right renal artery, for this reason, the left renal artery is susceptible to hypercholesterolemia, and the right renal artery is susceptible to hypertension. Second. the starting position of right renal artery is usually slightly higher than that of left renal artery, and the pressure of blood vessel wall gradually decreases from the proximal side to the distal side, therefore, the starting pressure of right renal artery is slightly higher than that of left,blood vessel wall of the right starting position must bear more pressure, so the intima of this position is vulnerable to be damaged, in order to promote plaque formation. Third. The abdominal aorta is on the left, and the position of right kidney is lower than that of the left kidney, hence, the length of the right renal artery trunk is longer than that of the left and the renal artery angle A is smaller than the left one, consequently, blood hypercholesterolemia is vulnerable to deposit in the left renal artery. Finally, the degree of tortuosity of abdominal aorta affects local hemodynamics, which influence the formation of RAP. To the people with the above risk factors, the vessel wall is lack of flexibility and prone to be damaged, the bloodstream is low and the cholesterol is easy to deposit in the wall, which contribute to the formation of plaque [31].

There was no significant difference on the other factors between the two groups. The results are identical with previous researches [32].Our finding that there is no statistical difference on the incidence of RAP between men and women is consistent with previous studies.

### Anatomical factors

#### Distribution and type of RAP

The current study demonstrates that the probability of RAP from the left and right renal artery is similar. However, Zhun et al. [15] reported that the prevalence of left RAP was higher than that of the right RAP. The deviation is mainly concerned about the population of limited sample, regional differences from sample, differences in baseline diseases and the different phases of RAP.

The atherosclerotic plaques are the most prone to appear at the proximal sections, then, the middle and distal sections of renal artery, diffuse distribution is the rarest. The above outcome is correspond with the conclusions of Tafuri et al. [33]. The proximal segment is the most vulnerable to suffer the impact of blood flow velocity and fluid shear stress (FSS), and the vascular intima is damaged more easily, vascular smooth muscle cells are prone to differentiate into an osteoblast/chondrocyte phenotype, where inflammatory mediators, recruitment of activated macrophages, lipids and inflammatory factors are more likely to release and deposit [34]. The above factors are conducive to provoke and regulate the pathological process of plaque.

Among all plaques, calcified plaques are the most common ones, followed by mixed plaques, non-calcified plaques are the least. The possible causes are as follows: the metabolic regulation mechanism of RAP that oxidized lipids promote calcification in these vascular cells, the process of systemic atherosclerosis, the appearance time on calcification of plaque and the decrease of glomerular filtration rate [35]. In addition, calcified plaques and mixed plaques are prone to be found because of being more apparent contrast with surrounding tissues than non-calcified plaques. In the early stage of RAP, RAS is slight with positive remodeling of the lumen, and it rarely caused clinical symptoms (such as renal hypertension and renal artery insufficiency syndrome and so on). Therefore, there are fewer imaging examinations to be done, and the corresponding detection rate is relatively low [36]. RAP is strongly related to calcium deposition in the coronary, carotid, infra-renal aorta and common iliac arteries [7]. Hence, it can indirectly predict the probability of RAP through the demonstration of artery plaque from other main artery to make suitable treatment strategy [37].

#### Anatomical factors of RAP

This study indicates that the RAP is significantly correlated with the renal angle A, which is in accordance with a previous study [13].When the renal artery angle A is greater, the flow from regions of the proximal sections with bifurcations or curvatures and the downstream are more prone to appear complex changes in direction and the blood FSS is greater, which is more prone to damage the vascular intima and activate FSS pathways (shear-responsive kinases, GTP-ases, ion channels, and other signaling molecules and signaling events(including inflammatory mediator), as well as many downstream genes and micro-RNAs and so on), the process of lipid deposition, inflammatory activation and thrombosis is more prone to be triggered. Finally, these factors facilitate the formation of plaque [38–40]. However, the renal artery angle A makes no significant impact on concrete distribution, type and stability of RAP, which is related to the above pathophysiology.

The angle B or the rise of the most curved arch make no noticeable impact on RAP. The possible reasons are as follows: First. The renal artery was straight in some cases. Second. The measurement deviation is displayed in view of the shorter renal artery trunk. Third. The method of evaluation has limitations. Finally. The number of selected samples is relatively few. The RAP is not linked with the diameter of the proximal/middle/distal /average of the renal artery, which is unanimous with the results of previous study. The latter suggested that there was no apparent correlation between RAP and the lumen diameter. The reasons are related to the positive remodeling of the plaque, vessel lumen self-regulation mechanisms. The length of the main renal artery may not be a primary contributor to form plaque despite affecting the passing time of the main blood flow, which is consistent with the former literature. The distance between the left and right renal artery initial positions does not affect the blood flow of the renal artery trunk. Consequently, it is not apparent correlated with RAP. The accessory renal artery and premature branch of the renal artery are the common mutations of the renal artery, which are not associated with the formation of RAP.

The present study makes a systematic and comprehensive assessment on factors related with RAP, but it still exists some limitations. First of all, the sample size of this study is relatively small, and it is still necessary to increase the sample size for further verification of the results in the future. Secondly, the relationship between the specific location on the cross section of the plaque and RAP was not further explored in depth. Thirdly, whether MRI functional imaging can be used to evaluate the correlation between plaque and relevant influencing factors or not, which need to be further studied in the future [41–43]. Finally, the plaque was analyzed only from CT images, not combined with IVUS, which may be considered as the direction of future research.

## Conclusion

In conclusion, right RAP is significantly related to age, hypertension and right renal artery angle A and left RAP is related to age, hypercholesterolemia and left renal artery angle A. Among all influence factors, only the renal artery angle A, age, hypertension and hypercholesterolemia are correlated with RAP. It can be a good precaution that those with the above factors are prone to appear RAP in clinical practice. It is beneficial for risk stratification of RAP, secondary prevention of RAP. Furthermore, it is conducive to select donor of kidney transplant. In addition, it is useful for the treatment guidance and post-treatment assessment of renal artery denervation [44]. The current study makes a systematic and comprehensive assessment about RAP, which lays a foundation for the relevant study of RAP. But this study also has forging limitations, which need to be further researched in the future.

## Data Availability

The data that support the findings of this study are available on request from the corresponding author.
